# Mosaic Genome of a British Cider Yeast

**DOI:** 10.3390/ijms241311232

**Published:** 2023-07-07

**Authors:** Beatrice Bernardi, Florian Michling, Jürgen Fröhlich, Jürgen Wendland

**Affiliations:** 1 Department of Microbiology and Biochemistry, Hochschule Geisenheim University, Von-Lade-Strasse 1, 65366 Geisenheim, Germany; beatricebernardipt@gmail.com (B.B.); florian.michling@hs-gm.de (F.M.); 2 Geisenheim Yeast Breeding Center, Hochschule Geisenheim University, Von-Lade-Strasse 1, 65366 Geisenheim, Germany; 3 Erbslöh Geisenheim GmbH, 65366 Geisenheim, Germany; dr.juergen.froehlich@outlook.com

**Keywords:** fermentation, domestication, hybrid, *Saccharomyces*, volatile aroma compounds, next-generation sequencing

## Abstract

Hybrid formation and introgressions had a profound impact on fermentative yeasts domesticated for beer, wine and cider fermentations. Here we provide a comparative genomic analysis of a British cider yeast isolate (E1) and characterize its fermentation properties. E1 has a *Saccharomyces uvarum* genome into which ~102 kb of *S. eubayanus* DNA were introgressed that replaced the endogenous homologous 55 genes of chromosome XIV between *YNL182C* and *YNL239W*. Sequence analyses indicated that the DNA donor was either a lager yeast or a yet unidentified *S. eubayanus* ancestor. Interestingly, a second introgression event added ~66 kb of DNA from *Torulaspora microellipsoides* to the left telomere of *SuCHRX*. This region bears high similarity with the previously described region C introgression in the wine yeast EC1118. Within this region *FOT1* and *FOT2* encode two oligopeptide transporters that promote improved nitrogen uptake from grape must in E1, as was reported for EC1118. Comparative laboratory scale grape must fermentations between the E1 and EC1118 indicated beneficial traits of faster consumption of total sugars and higher glycerol production but low acetic acid and reduced ethanol content. Importantly, the cider yeast strain produced high levels of fruity ester, including phenylethyl and isoamyl acetate.

## 1. Introduction

*Saccharomyces uvarum* (also known as *S. bayanus* var. *uvarum*) is the most distantly related species to *S. cerevisiae* in the *Saccharomyces* genus [1]. It is also the sub-genome donor of *S. bayanus* (*S. cerevisiae* × *S. eubayanus* × *S. uvarum*) [2]. *S. uvarum* is a psychrophilic yeast associated mainly with cider and white wine fermentations in northern wine regions, especially France, Hungary, northern Spain, and Canada [3,4,5,6]. Additionally, *S. uvarum* is used in the production of the red dry wine Amarone (Valpolicella, Italy) and the fermentation of apple chicha in Patagonia [7,8].

The sensory properties of wines are impacted by yeast secondary metabolites released during fermentation and the wine-making process. The aroma profiles of *S. uvarum* differed from those of *S. cerevisiae* as *S. uvarum* generated larger amounts of higher alcohols (particularly isoamyl alcohol, phenyl ethanol and their respective esters) during grape must fermentation [9,10,11]. In *S. uvarum*, succinic acid produced during fermentation can contribute to an increase in total acidity of the wine. In addition, acetic acid, which has a negative organoleptic impact on wine, is produced in lower quantities compared to *S. cerevisiae* [12]. Furthermore, due to an increase in glycerol production, the final ethanol content of *S. uvarum* fermentations was slightly lower than that of *S. cerevisiae* fermentations [13,14,15,16,17,18,19].

Regarding the natural geographical distribution of *S. uvarum*, four populations were distinguished: two South American (SA-A and SA-B), the Holarctic (H) and the Australasia. SA-A/B strains were found to be associated with *Nothofagus* (southern beech), in Patagonia, while most of the wild isolates of the Holarctic group were found in relationship with oak trees in North America. Several isolates from cider and wine fermentations were found in Europe and these strains clustered with the Holarctic population. The Australasia population, with most of the isolates coming from New Zealand, was determined to be the most distantly related to the other groups [14,20]. Moreover, an admixed population (H/SA-A) was reported from Patagonia and its origin was proposed to have resulted from secondary contact after the introduction of apple trees in Argentina by European immigrants during the 19th century [14].

Hybridization, horizontal gene transfer and interspecific DNA introgression with subsequent genome rearrangements and adaptations are main mechanisms of genome evolution in domesticated species [21,22,23,24]. Hybrid formation in the genus *Saccharomyces* is quite frequent as there is no prezygotic barrier within this genus [25,26]. The best known and most successful hybrids in the fermentation industry are lager yeasts, which are hybrids between *S. cerevisiae* and *S. eubayanus* [1,27]. Most of the *S. uvarum* Holarctic isolates from industrial environments possessed DNA introgression from two *Saccharomyces* species, *S. eubayanus* and *S. kudriavzevii*. Moreover, the impact of human domestication is demonstrated by conserved patterns of inheritance found in populations of isolates from the same industrial environments. Here, *S. eubayanus* DNA was found to be present in *S. uvarum* strains isolated from cider fermentations [28].

In the widely used wine yeast EC1118 three interspecific DNA introgressions linked to important traits of wine fermentation were found [29]. These DNA regions (A, B and C) originated from different species and are found at different chromosomal locations in the EC1118 genome. Region A (38 kb) is located at the left sub telomeric region of chromosome VI; region B (17 kb) was acquired by horizontal gene transfer from *Zygosaccharomyces bailii* and it is present in three copies on chromosome XIV, XII and X [30]. This region was identified in up to 4 copies in 28 different wine strains. Region C was found in EC1118 and other wine yeast strains and apparently evolved by reduction of an original 165 kb DNA-fragment of *Torulaspora microellipsoides* [31]. In EC1118, a single copy of a 65 kb fragment of region C is located at the subtelomeric region of the right arm of chromosome XV. This region harbors genes advantageous to wine fermentation, e.g., sugar and oligopeptide transporter genes, particularly the *FOT1* and *FOT2* fungal oligopeptide transporter genes [31,32].

In moderate climate zones, apple is one of the most important fruits, which is either consumed directly, pressed into apple juice or fermented into apple wine and cider. Traditionally, freshly pressed juice is fermented spontaneously, while industrial apple wine and cider production often uses apple juice concentrate and relies on dry yeast starter cultures as performed in wine fermentations. Major differences between apple juice and grape must are the sugar and organic acid compositions [33]. Grape must contains equal amounts of glucose and fructose. In contrast, apple juice additionally contains sucrose and the ratios of fructose:glucose:sucrose are close to 3:1:1 [34]. Hence, the sugar composition of apple juice may require a more fructophilic yeast to complete fermentation. The dominant acid in apple juice is, of course, malic acid, while grape must contains malic acid and tartaric acid.

Similar to white wine production, low temperature fermentation is preferred in apple wine production to generate a richer ester profile and preserve fresh fruity notes [35,36]. However, since *S. cerevisiae* is outcompeted at lower temperatures by more cold-tolerant *Saccharomyces* species, apple wine and cider fermentations often rely on strains formerly described as *S. bayanus* var. *uvarum* [20,37]. Additionally, contributions of *S. kudriavzevii* to both, wine- and cider-making have been described in recent years [38,39,40]. Similar to lager beer fermentations, low-temperature cider fermentations favor hybrids of *S. cerevisiae* with the cold tolerant *Saccharomyces* species *S. eubayanus*, *S. kudriavzevii* or *S. uvarum* [41,42,43].

In this study, we used comparative genomics and phenotypic analyses to characterize a cider yeast (E1), which was originally isolated from cider in Ross-on-Wye (Hereford, UK), from a winemaker’s point of view. We determined signs of adaptation/domestication, such as the introgression of region C into this strain that was confirmed to be an *S. uvarum* derivate. 

## 2. Results

We initiated this study to characterize potential European isolates of *S. eubayanus*. A successful isolation of *S. eubayanus* has in the meantime been reported from Ireland [44]. The E1 strain we report here was isolated from cider fermentations in Ross-on-Wye, Herefordshire, United-Kingdom. 

### 2.1. Genome Sequencing of E1, A British Cider Yeast

**Figure 1:**
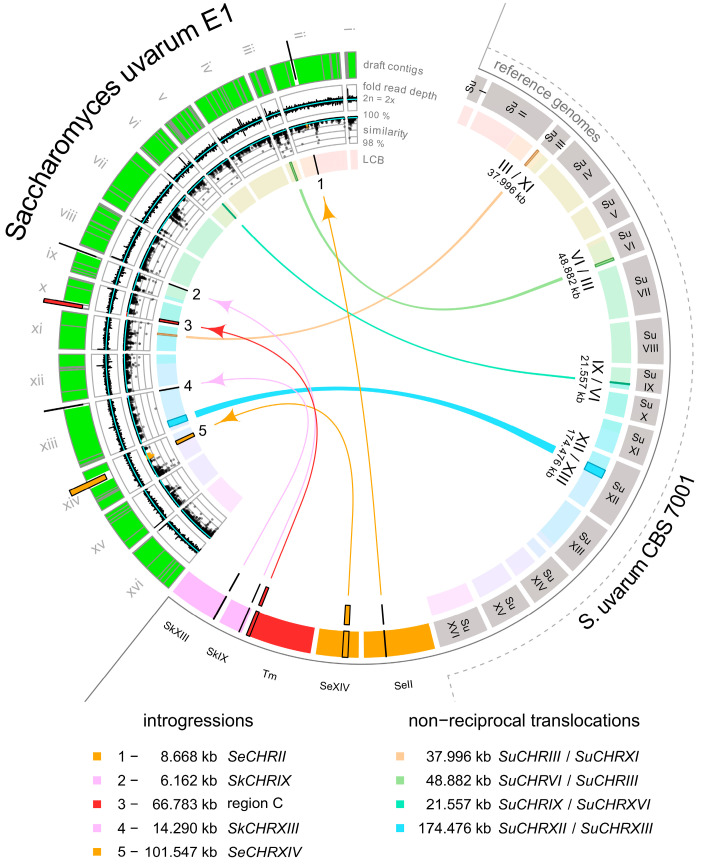
Non-reciprocal translocation and introgressions into the E1 genome. The four non-reciprocal translocations are shown as ribbons connecting segments found on one chromosome in CBS 7001 and translocated to a new position in E1, e.g., an approx. 38 kb fragment found on *CHRIII* in CBS 7001 was translocated to chromosome XI in E1. Five introgressions (1 to 5) are indicated as arrows originating from non-*Saccharomyces uvarum* source chromosomes and pointing to insertion sites in E1, e.g., for translocation 1: an 8.7 kb DNA fragment from *S. eubayanus* chromosome II was introgressed into E1 chromosome II. Fold read depth track is based on counting binned short reads aligned to the draft assembly. (Pairwise) Similarity track is based on Tamura and Nei two-parameter metric without γ correction [45] in consecutive 1 kb windows containing at least 900 aligned positions; only values >98% are shown (similarity was <98% in 147 of 11,419 windows). Cyan lines are median fold read depth and median similarity on the corresponding tracks. LCB track shows Locally Collinear Blocks between E1 draft genome and reference genome contigs. Reference genome contigs are in clockwise orientation and E1 contigs are oriented counterclockwise. %). Contig extent in the E1 draft genome is shown (grey subdivisions on green pseudochromosomes). *Su*—*Saccharomyces uvarum*; *Se*—*S. eubayanus*; *Sk*—*S. kudriavzevii*; *Tm—Torulaspora microellipsoides*; region C—region C from *T. microellipsoides*; and LCB—Locally Collinear Block (from Whole Genome Alignment).

**Figure 2:**
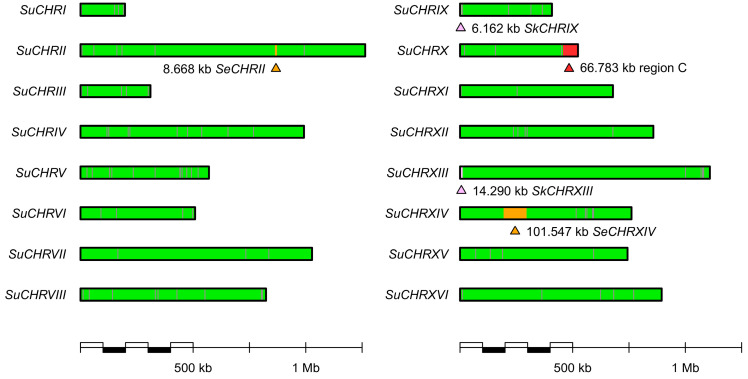
*Saccharomyces uvarum* E1 genome structure and the positions of five introgressed, non-*Saccharomyces uvarum* DNA fragments. The E1 contigs were aligned to the 16 chromosomes of CBS 7001. E1 is a diploid yeast strain, and all introgressions were also present in both of the respective chromosomes. The positions of introgressions are marked by a triangle. *Su*—*Saccharomyces uvarum*; *Se*—*S. eubayanus*; *Sk*—*S. kudriavzevii*; and region C represents the region C from *Torulaspora microellipsoides*.

**Figure 3:**
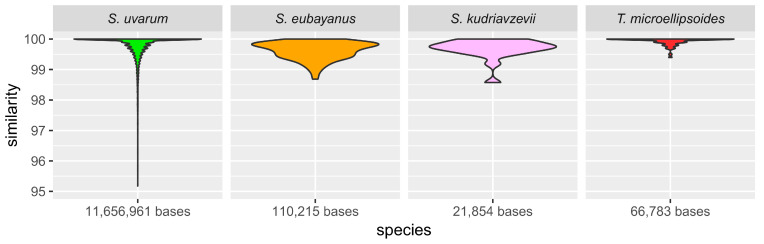
Distribution of similarity of *Saccharomyces uvarum* E1 DNA with the respective reference genomes. Similarity was calculated for consecutive 1 kb windows containing at least 900 aligned positions (Tamura and Nei two-parameter similarity without γ correction [46]). Lower extent of vertical axis (similarity) was restricted to only display values > 95% (similarity < 95% was observed only in a total of 23 windows). The number of bases derived from each species is listed at the bottom.

We used next-generation sequencing platforms to assemble the draft genome of E1. To this end two libraries were used. One consisted of an Illumina MiSeq platform with 2 × 300 bp paired-end reads while the other used a NextSeq500 platform with 2 × 150 bp paired-end reads (Table 1). Both datasets were assembled into a 12 Mb genome. Blast analyses revealed that E1 is an *S. uvarum* strain based on its close similarity to the *S. uvarum* type strain, CBS 7001. The CBS 7001 genome was assembled into 16 chromosomal and 1 mitochondrial contig [45]. We aligned the E1 sequences to this assembly and found overall well aligning sequences to CBS 7001 with several notable exceptions. Among them were four major translocation events (considering DNA fragments > 5 kb). These moved ~282 kb of *S. uvarum* DNA via non-reciprocal translocations to new locations in E1 (Figure 1). The mitochondrial genome of E1 was also found to be highly similar to the *S. uvarum* mitochondrial genome of CBS 7001. Furthermore, the E1 genome harbors five locations indicative of DNA-introgressions, some only containing a few genes but also two extended regions. Based on DNA-identities, the original donors of these introgressions were identified as *S. kudriavzevii, S. eubayanus* and *Torulaspora microellipsoides* (Figure 2 and Figure 3, Table 2).

### 2.2. Analysis of the Introgressed Region Derived from S. kudriavzevii

Two small introgressed regions were derived from *S. kudriavzevii*. These encompass 6.1 kb and 14.3 kb, respectively. On the 6.1 kb fragment, a partial *SUC2* gene was found, the *S. kudriavzevii* genes *SkSMU2*, *SkPOT1* and a chimeric *BNR1* gene generated by the introgression. The 14.3 kb introgression was assembled from three E1 contigs. On this fragment, nine genes were found: *SkARR3* (a plasma membrane antiporter involved in arsenic resistance), *SkARR2* (an arsenate reductase required for arsenate resistance), *ARR1* (a transcriptional regulator of genes involved in resistance to arsenic compounds), *SkYHL044W*, *SkYML131W*, *SkERO1*, *SkCOX14*, *SkMSC1* and a chimeric *RSC9* generated by the introgression.

### 2.3. Analysis on the Origin of S. eubayanus DNA Introgressed into the E1 Cider Yeast

Two introgressions originated from *S. eubayanus*. A smaller fragment of 8.7 kb located on E1 *CHRII* harbors seven genes: *SeSWA2* (chimeric-fusion site), *SeDAD4*, *SeASP1*, *SeMRPL35*, *SeTIM11*, *SePEP7* and *SeUTP4* (chimeric-fusion site). Of these genes, *ASP1* (*YDR321W*) is of special interest. It encodes an L-asparaginase that catalyzes the hydrolysis of asparagine to aspartic acid releasing ammonia [47]. This could be of adaptive value in cider yeasts as asparagine is an abundant amino acid in apple must [48,49].

A large, 101.547 kb, introgression of *S. eubayanus* DNA into E1 was found on *CHRXIV* consisting of 53 genes between *YNL182C* and *YNL239W*. This introgression resulted in the replacement of the syntenic endogenous genes. The adjacent genes, *YNL181W* (*PBR1*) and *YNL240C* (*NAR1*) are chimeric genes generated by the introgression.

**Figure 4:**
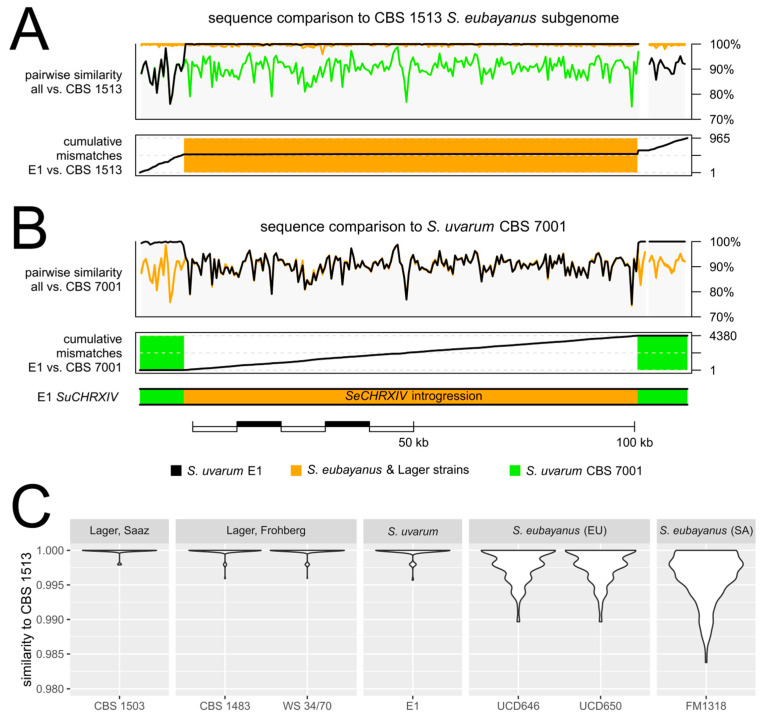
Large introgression of *S. eubayanus CHRXIV* DNA into E1. DNA sequence identity of the 102 kb *S. eubayanus* introgression into E1 to Saaz group lager yeast *S. eubayanus* subgenomes (**A**) and *S. uvarum* CBS 7001 (**B**) and sequence similarity of the 102 kb *S. eubayanus* insert to natural *S. eubayanus* strains or industrial hybrids (**C**). Pairwise similarity was calculated for 500 nt consecutive windows. Cumulative mismatch plots illustrate the origin of the introgressed segment; i.e., few mismatches accumulated along the aligned E1 DNA sequence when compared to a DNA sequence conspecific to the introgression donor. Black line—sequence similarity E1/CBS 1513 or E1/CBS 7001 (panels A and B, respectively); orange line—sequence similarity E1/other lager yeasts; green line (panel B)—E1/CBS 7001.

DNA sequence comparison of the introgressed segment showed the number of mismatches between E1 and the homologous sequences from multiple lager yeasts’ *S. eubayanus* subgenomes was approx. 10-fold lower than the number of mismatches between E1 and newly discovered European pure *S. eubayanus* isolates UCD 646 and UCD 650 (Table 3; Figure 4C [44]). The number of mismatches among lager yeasts was approx. one half of that between E1 and lager yeasts. Similar introgression events regarding *CHRXIV* of *S. uvarum* have previously been described for other *S. uvarum* strains (see Discussion). We therefore propose the introgressed *S. eubayanus* DNA was originally acquired by *S. uvarum* from a pure *S. eubayanus* donor with very high sequence similarity to the unknown lager yeast *S. eubayanus* subgenome donor. It is, however, also possible that individual introgressions of *S. eubayanus* DNA were sourced from lager yeast (*S. cerevisiae* × *S. eubayanus*) directly.

### 2.4. Analysis of the Introgressed Region Derived from T. microellipsoides

**Figure 5:**
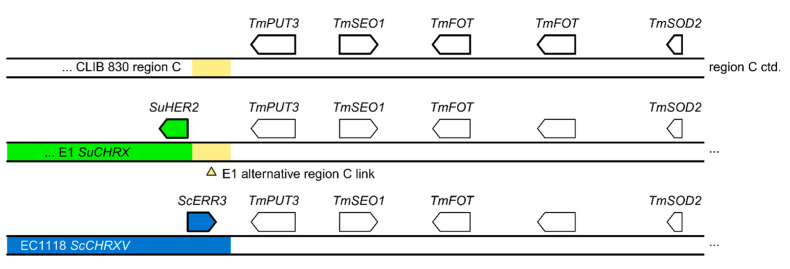
E1 region C variant. The *T. microellipsoides* (*Tm*) introgression into E1 contains an additional 1785 nt *Tm*-DNA missing in the wine strain EC1118, from which region C was first described. *Su*—*Saccharomyces uvarum*; *Sc*—*S. cerevisiae.* Genes and their transcriptional orientation of the 5′-part of region C are indicated by arrows.

Interestingly, E1 also harbored a DNA segment that has been described as region C in the wine yeast EC1118 [29]. This segment originated from *T. microellipsoides* and was found at the left telomere of E1 *CHRX*. This segment was found to contain the entire region C introgression described in EC1118 (Figure 5).

Region C in E1 was assembled in two contigs (NODE_53 and NODE_81) covering a total of 66.8 kb. A closer comparison of the E1 sequence and region C from EC1118 with *T. microellipsoides* showed an additional sequence of 1785 bp, which is not present in EC1118. This 1.8 kb fragment contains 316 bases of the 5′ end of the *HXT2* ORF. On this E1 contig upstream of the 1.8 kb, sequences with high similarity to *CHRX* of *S. uvarum* CBS 7001 were found, indicating that this region is positioned at the left telomere of E1 *CHRX*. 

In EC1118, region C contains 19 genes and approximately 600 bp of *ARB1*, which is located ~92 kb upstream of the region C ‘core’, thus indicating a large gap compared to the *T. microellipsoides* genome sequence (as previously reported by Marsit et al. [31]). The *ARB1* gene sequence is not present in E1, but it was found in other domesticated wine yeast isolates [31]. 

### 2.5. Characterization of Growth and Fermentation Performance of E1

Growth assays were used to compare the E1 cider yeast strain with the wine yeast EC1118, *S. uvarum* CBS 7001 and the lager yeast *S. carlsbergensis*. At 10 °C growth of *S. uvarum* CBS 7001 and E1 was substantially better than that of EC1118 after two days of incubation. Similar results were obtained on solid media supplemented with menadione, inducing oxidative stress (Figure S1). 

**Figure 6:**
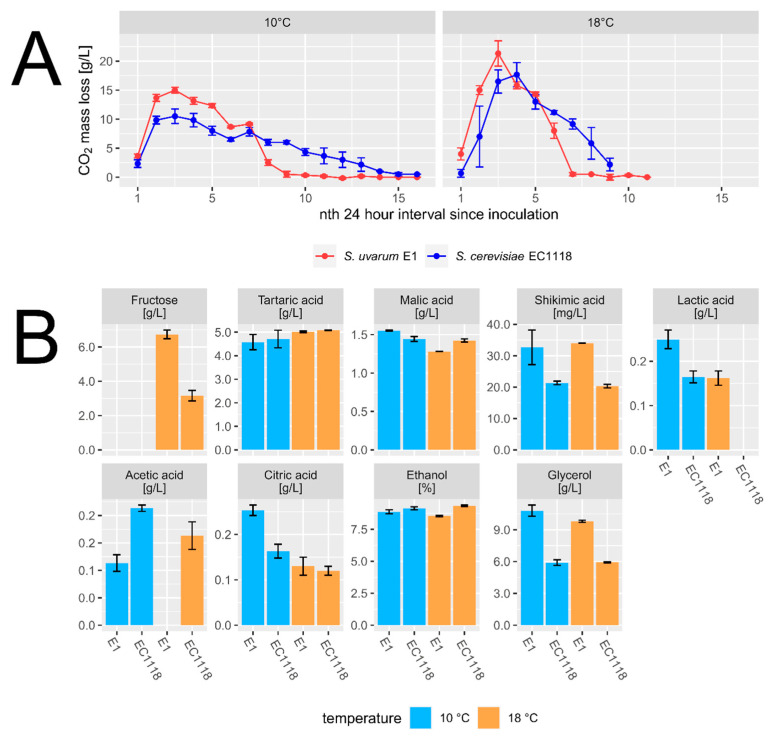
Comparative fermentations between the cider yeast strain and EC1118. Müller-Thurgau must was fermented at either 10 °C (**A**) or 18 °C (**B**) by the cider yeast E1 (red lines) or the wine yeast EC1118 (blue). CO_2_ release was measured by monitoring daily mass loss of the fermentation vessels. HPLC analysis of ethanol, glycerol and acetic acid are shown.

Fermentation performance of the cider yeast strain was evaluated with lab-scale fermentation assays using Müller-Thurgau grape must at different temperatures (18 °C and 10 °C). Compared to EC1118, the E1 cider strain fermented faster at both temperatures (Figure 6). All strains fermented to dryness and completely metabolized the available glucose and fructose. Glycerol production was substantially higher (*p* < 0.01) in E1 (~10 g/L) compared to EC1118 (~6 g/L), which can, at least in part, explain the reduced alcohol production of E1. Interestingly, acetic acid production was significantly higher in EC1118 (Figure 6). Cell counts at the end of fermentation at 18 °C indicated that the *S. uvarum* strain reached higher cell densities (5 × 10^7^) compared to EC1118 (2.8 × 10^7^). While E1 fermentations increased in cell numbers, EC1118 cell numbers decreased towards the end of fermentation. Additionally, petite cells were only observed in EC1118 at the end of fermentations but not in E1 (our unpublished data).

**Figure 7:**
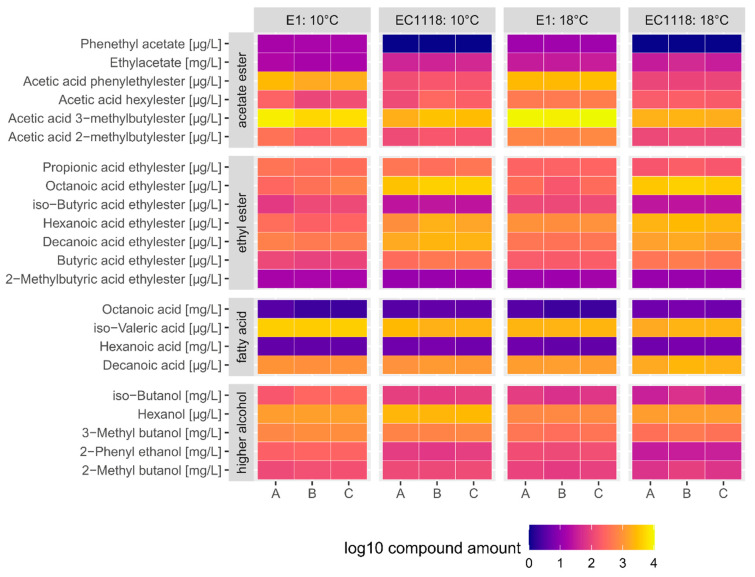
Volatile aroma compounds (VOCs) production. At the end of fermentation, compounds produced by E1 and EC1118 were analyzed by GS/MS. Fermentations were performed in triplicate (A,B,C) at 10 °C and 18 °C. Concentrations of VOCs produced are normalized to logarithmic scale and converted into a color-coded heat map indicating consistency of fermentations and allowing for better comparison between E1 and EC1118.

The formation of volatile aroma compounds (VOCs) was analyzed at the end of fermentation. We found an increased production of higher alcohols (e.g., 2-phenyl ethanol) in E1 compared to EC1118. The cider yeast strain also produced higher ester levels, e.g., phenethyl acetate as well as the fruity acetate ester isoamyl acetate (Figure 7).

### 2.6. Deletion of FOT Genes in E1 Reduces Nitrogen Uptake

**Figure 8:**
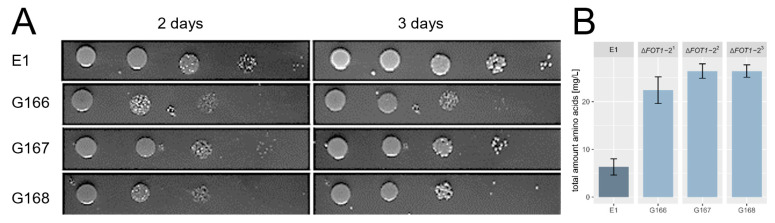
Growth and N assimilation by *fot1/fot2* deletion strains. (**A**) Growth of the *fot1/fot2* deletion strains (G166–G168) on beer plates was compared to the E1 wild type strain. Images were obtained after two and three days of growth at 25 °C. (**B**) Amino nitrogen content at the end of fermentation with the *fot1/fot2* deletion strains was compared to E1. Error bars indicate standard deviation.

In order to characterize a potential benefit of the *T. microellipsoides* introgression of *FOT* genes into E1, we deleted both *FOT1* and *FOT2* alleles of this strain by consecutive rounds of gene-targeting. Deletion strains were verified by PCR that indicated the absence of *FOT* genes in three independent mutants (Figure S2). Two comparative growth tests were performed with these strains. First, E1 and the *FOT*-deletion mutants were grown on beer plates that contained finished beer as a sole nutrient source. This indicated weaker growth of the *FOT* mutants compared to E1 (Figure 8A). Second, at the end of grape must fermentation, the total amino acid content was measured to quantify amino acid nitrogen utilization of these strains. This revealed amino acid uptake deficiencies of the *FOT* mutants compared to E1 (Figure 8B). On average, E1 was able to consume 98% of the total initial amino acids present in grape must (375.7 mg/L), while the mutant strains assimilated only 93% free amino nitrogen.

## 3. Discussion

The domestication of yeast strains for beer and wine fermentations progressed in specific man-made fermentation niches that led to a remarkable evolution of the microorganism’s genomes. Interspecies hybridizations, DNA introgressions and horizontal gene transfers and genomic rearrangements, have been linked to selective environmental pressures such as limited nutrient conditions, high sugar concentrations and elevated ethanol level [22,50,51].

In this study, we sequenced, and de novo assembled the genome of a cider yeast isolate, E1. The isolate was identified as an *S. uvarum* strain harboring four major translocation and two DNA introgression events. Approximately 102 kb of *S. eubayanus* DNA were located on chromosome XIV. Analysis of mismatches suggested this DNA fragment was acquired from a yet unknown pure *S. eubayanus* strain, different from the recently reported European wild type *S. eubayanus* strain(s) [44], however, likely closely related to the lager yeast *S. eubayanus* subgenome donor.

Previously, the introgression of *S. eubayanus* DNA into *S. uvarum* had been analyzed, and strains with more extensive DNA contributions from *S. eubayanus* were found [20,28]. It was noted that these introgressions were found predominantly in strains from the Northern hemisphere associated with human fermentation activities. In E1, 54 *S. uvarum* genes were lost and replaced by their *S. eubayanus* homologs. Several of these genes are essential (*CSL4*, *IPI3*, *KAR1*, *POP1*, *RAP1*, *RIO2* and *SSU72*), required for cell-size control (*WHI3*) or for general gene regulation (*GCR2*, *RAP1* and *URE2*). Other *S. uvarum* strains with *CHRXIV* introgressions of *S. eubayanus* DNA covered a similar region. It would, therefore, be interesting to determine the adaptive value of these genes in cider fermentations. With the lager yeast hybrid of *S. cerevisiae* and *S. eubayanus* it was argued that the contribution of *S. eubayanus* to the hybrid was the cold fermentation ability [52]. However, *S. uvarum* is psychrophilic on its own. Apparently, hybridizations of *S. eubayanus* into *S. uvarum* are not very successful on the genome scale and left us with the now observable introgression events. Similar evolutionary trajectories of hybrid lineages have been reported in olive populations between *S. cerevisiae* and *S. paradoxus*. Initially homoploid/diploid hybrid genomes then evolved by massive losses within the *S. paradoxus* subgenome [53]. 

An addition of genes into the E1 genome resulted from the introgression of ~66 kb of region C-like DNA located on chromosome X. Based on the presence of an approx. 2 kb DNA segment observed in *T. microellipsoides* but missing in EC1118, and assuming that after the initial horizontal event region C evolution in *Saccharomyces* was largely governed by segmental loss, *S. uvarum* may have acquired region C early in region C evolution in *Saccharomyces* or from a strain other than EC1118. 

DNA introgression events in wine yeasts could be adaptive to increase the fitness under fermentation conditions, i.e., be the result of domestication. Key genes of the *T. microellipsoides* introgression are the *FOT* genes. *FOT* genes encode oligopeptide transporters which broaden the range of oligopeptides that yeasts can use during fermentation of grape and apple musts [31]. Deletion of all four *FOT* alleles in E1 revealed a deficiency in growth on limited nitrogen media and reduced assimilation of amino nitrogen from must compared to the E1 parental strain. Deletion mutants of E1 lacking *FOT* genes showed less uptake of glutamate, serine, alanine and cysteine. FOT transporters were shown to have a high specificity to di- and tripeptides containing glutamate [54]. Interestingly, within the 54 *S. eubayanus* genes E1, *NPR1*, which encodes a protein kinase that prevents ubiquitin-mediated proteolysis of amino acid transporters, could improve nitrogen uptake during wine fermentation [55,56]. 

*S. uvarum* E1 domestication resulted in better fermentation performance and distinguished flavor output compared to the EC1118 wine yeast in laboratory small scale fermentations. Particularly, lower acetic acid production and enhanced glycerol production (by up to 50%, resulting in reduced ethanol levels of E1 by up to 8%) in comparison to EC1118 represent favorable traits. With climate change and consequent increased sugar amounts in grape musts, alcoholic fermentations result either in sweeter wines or in increased ethanol content of dry wines. This drives the search for alternatives to achieve alcohol reductions either by chemical means or by alternative yeast strains. E1 shows some potential in this area, which is further enhanced by the increased production of fruity esters.

Taken together, *S. uvarum* strains with specific introgressions as identified in E1 and others may provide useful additions to the repertoire of wine yeast starter cultures.

## 4. Materials and Methods

### 4.1. Strains Used and Generated

The strains constructed and used in this study are listed in Table S1. The strains used for genomic data analysis are listed in Table S2.

### 4.2. Media, Growth and Fermentation Conditions

The strains were cultivated in YPD (1% yeast extract, 2% bacterial peptone and 2% glucose). YPG (1% yeast extract, 2% bacterial peptone and 2% glycerol) and YPGD (0.1% yeast extract, 1% bacterial peptone, 0.1% glucose and 2% glycerol) agar plates were used for petite screening and prepared according to Petersen et al. [57]. Beer plates were prepared with commercial pilsner beer solidified with 2% agar as in all solid media. YPD plates were supplemented with 10 µg/mL of menadione for stress tolerance characterization to reactive oxygen species (ROS). For recombinant strain construction, YPD plates were supplemented with geneticin/G418 (200 µg/mL) or nourseothricin/clonNAT (50 µg/mL).

Lab-scale fermentations were carried out in triplicates with strains precultured in YPD. Fermentations were inoculated with 10^7^ cell/mL. Müller-Thurgau grape must with 51.5 mg/L amino acid nitrogen and 43 mg/L free ammonium (total yeast available nitrogen: 94.5 mg/L) was used for all fermentations. Bench top fermentations (200 mL of total volume) were run on magnetic stirrer platforms in cabinets at 18 °C and 10° C. The fermentation progress was monitored by daily weight loss measurements.

For spot assays, strains were grown overnight in YPD at 25 °C. Tenfold serial dilutions were prepared and 5 µL of each dilution were spotted on plates. To assay growth at low temperature, plates were incubated at 10 °C for up to three days.

### 4.3. FOT-Gene Deletions

Yeast cells were transformed with the high efficiency LiAc/single strand carrier DNA/Polyethylene glycol method with a heat shock of 42° for 15 min [58]. Yeast cells were transformed with PCR-generated cassettes bearing short-flanking homology regions to the target locus and confirmation of correct deletion was conducted by diagnostic PCR as described previously [59]. The primers used are listed in Table S3. E1 is a diploid yeast strain. Thus, two rounds of PCR-based gene targeting were performed to delete both alleles of *FOT1* and *FOT2* using two dominant marker genes *YES1* (provides G418-resistance) and *YES3* (provides clonNAT-resistance) [60]. Generated mutants grew in the presence of both antibiotics, had correctly integrated both marker genes and had lost *FOT1* and *FOT2* ORFs (Figure S2). 

### 4.4. Analytical Methods

Chemical analyses were carried out as described previously [61]. Residual sugars and organic acids content after fermentation were analyzed by high-pressure liquid chromatography (HPLC) on an Agilent 1100 Series column (Agilent Technologies, Waldbronn, Germany). Residual yeast assimilable nitrogen (YAN) after fermentation was detected with primary amino acid (NOPA) measurement combined with ammonia determination (Megazyme, Bray, Ireland). Volatile aroma compounds were analyzed by gas chromatography using a GC 7890A (Agilent, Santa Clara, CA, USA), coupled with MSD 5977B mass spectrometer (Agilent, SantaClara, CA, USA).

Residual amino acid content at the end of the fermentation was detected by post- column derivatization with ninhydrin and the detection at 440 nm and 570 nm using maintenance-free LED photometers with ARACUS amino acid analyzer (membraPure GmbH, Hennigsdorf, Germany). Sample preparations and analyses were performed as described previously [62].

### 4.5. Genome Sequencing and Assembly

DNA-extraction, library generation and next generation sequencing were carried out by LGC Genomics GmbH (Berlin, Germany) and GenXPro GmbH (Frankfurt am Main, Germany). Read quality was evaluated using FastQC version 0.11.8 (https://github.com/s-andrews/FastQC). Read preprocessing and quality trimming were conducted with software tools provided by the BBTools software suite, version 38.84 (following guidelines from DOE Joint Genome Institute (https://jgi.doe.gov/data-and-tools/bbtools/bb-tools-user-guide/bbmap-guide/)) [63]. De novo assembly was performed using SPAdes version 3.14.1 [64] in only-assembler mode, in single runs, for odd-length kmer ranging from 27 to 249 value and in a single run for the same k-mer value. For draft genome assembly, the pre-processing trimming included: adapter clipping, removal of optical duplicates, removal of low-quality tiles and sequencing artifacts/spike-ins (e.g., PhiX), left-hand and right-hand quality trimming (to an average Phred score of 28) and removal of reads below 41 bases. More detailed bioinformatics methods are compiled in a Supplementary Note.

## Figures and Tables

**Table 1:** Genome Sequencing of E1.

Strain	E1 Cider Yeast (ROW 169)
Library No. 1	2 × 300 bp paired-end, 724,688 read pairs
Library No. 2	2 × 150 bp paired-end, 1,971,578 read pairs
No. of read pairs	2,696,266
Genome size	11,987,464 bp
No. of scaffolds	252
Scaffold N50	15
Scaffold L50	236,996 bp
Largest scaffold	1,000,723 bp
GC content	40%

**Table 2:** Introgressions into the *Saccharomyces uvarum* E1 genome.

Source	Size [bp]	Position in E1	DNA Identity	N	Genes	E1 Contigs
*S. eubayanus* *CHRII*	8668	*CHRII*	99.70% ± 0.27%	17	7	1
*S. eubayanus* *CHRXIV*	101,547	*CHRXIV*	99.62% ± 0.36%	202	53	1
*S. kudriavzevii* *CHRXIII*	6162	*CHRIX*	99.84% ± 0.18%	10	3	1
*S. kudriavzevii* *CHRXIII*	14,290	*CHRXIII*	99.67% ± 0.23%	23	9	3
*T. microellipsoides*	66,783	*CHRX*	99.59% ± 2.32%	127	19	2

**Table 3:** Comparison of E1 *S. eubayanus* DNA with other strains *.

Strain	Name	E1	CBS 1503	CBS 1513	CBS 1483	WS 34/70	UCD646	UCD650	FM1318
*S. uvarum*	E1	-	99.981	99.977	99.978	99.977	99.808	99.804	99.612
*Saaz*	CBS 1503	19	-	99.990	99.991	99.990	99.807	99.801	99.611
*Saaz*	CBS 1513	23	10	-	99.987	99.986	99.803	99.797	99.607
*Frohberg*	CBS 1483	22	9	13	-	99.997	99.804	99.798	99.608
*Frohberg*	WS 34/70	23	10	14	3	-	99.803	99.797	99.607
*S. eubayanus*	UCD646	192	193	197	196	197	-	99.966	99.596
*S eubayanus*	UCD650	196	199	203	202	203	34	-	99.600
*S. eubayanus*	FM1318	400	401	405	404	405	416	412	-

*: Alignment length: 102,635; positions (100,197 positions after stripping of gaps; E1 segment length: 101,547 nt); bottom half: number of pairwise single nucleotide mismatches; upper half: pairwise identity (in %).

## Data Availability

The E1 genome has been deposited under BioProject PRJNA970106 (BioProject).

## Supplementary Materials

The supporting information can be downloaded at: https://www.mdpi.com/article/10.3390/ijms241311232/s1. References [65,66,67,68,69,70,71,72] are cited in Supplementary Materials.
