# Supplementary material for: Mosaic Genome of a British Cider Yeast
Source: Int J Mol Sci. 2023 Jul 7;24(13):11232. doi: 10.3390/ijms241311232 (PMC10342233; doi:10.3390/ijms241311232)
Supplement: Supplementary file 1 [file ijms-24-11232-s001.zip › ijms-2486959 supplementary new.pdf]

Supplementary Materials

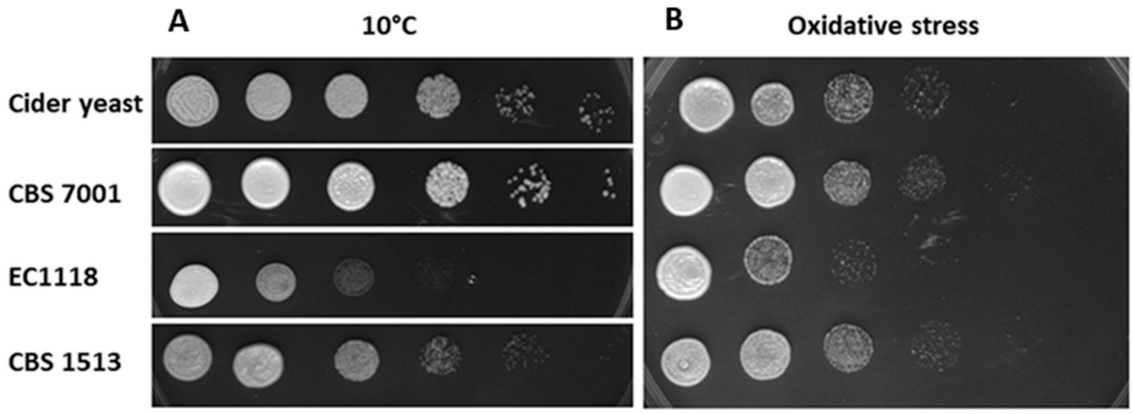

Figure S1. Growth assays. (A) The indicated strains were spotted in 10-fold serial dilutions on YPD solid media plates and incubated at 10°C for two days. (B) Strains were spotted on YPD plates supplemented with 10 μg/mL menadione and incubated at 25°C for three days.

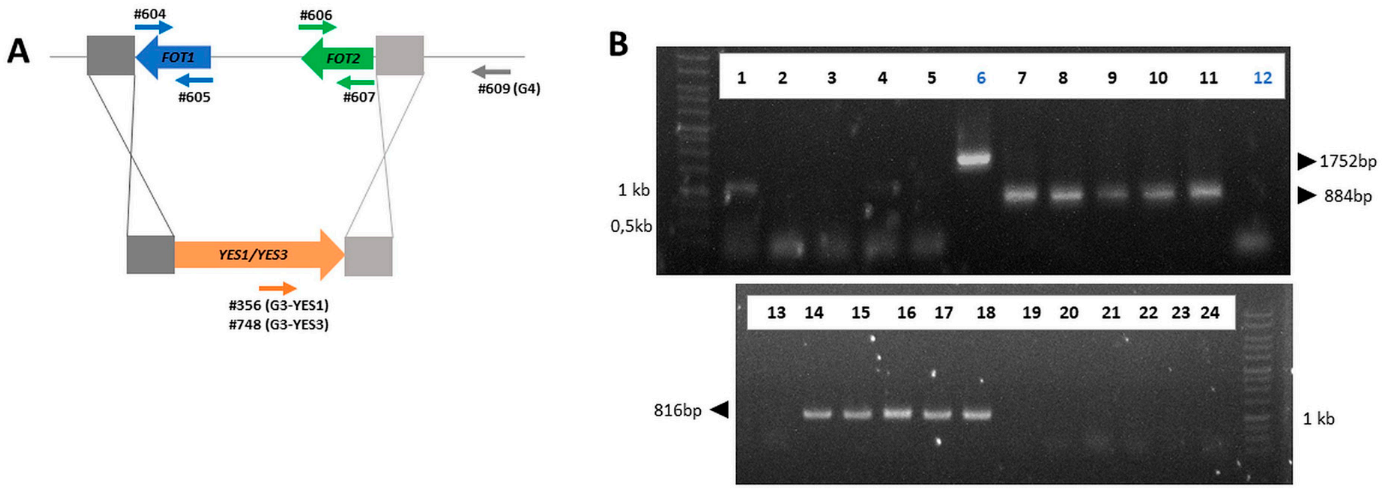

Figure S2. Construction of E1 FOT-deletion strains. (A) Schematic representation of the deletion strategy. To delete both alleles of *FOT1* and *FOT2*, sequential disruptions were achieved by replacing the target loci with *YES1* and *YES3*, respectively. (B) Gel electrophoresis images of diagnostic PCR to verify *FOT1*/*FOT2* deletions. Lanes **1-6**: *FOT1* PCR amplification with primer pair #604 and #605 (1752 bp); lanes **7-12**: diagnostic PCR indicating replacement of *FOT1*/*FOT2* by *YES1* with primer #356 and #609 (884bp). Lanes 6 and 12 are controls derived from E1; lane 1 shows a ~1kb band and thus this strain was excluded. Lanes **13-18**: diagnostic PCR indicating replacement of *FOT1*/*FOT2* by *YES3* (primer pair used: #748 and #609, fragment length was 816 bp. Lanes **19-24**: *FOT2* PCR amplification with primer #606 and #607 to detect any residual FOT gene DNA in the mutant strains. **Note**. With primer pairs #604/#605 and #606/#607 the presence or absence of *FOT1* and *FOT2* is tested, respectively. The PCR product generated with primers #356 and #609 indicates correct integration of the *YES1* marker; similarly, primers #748 and #609 indicate correct integration of the *YES3* marker. Three of the four verified *fot1*/*fot1*/*fot2*/*fot2* strains (G166, G167 and G168) were picked for further analysis.

Table S1. Strains used or constructed in this study.

Strain	Features/ genotype	Source
S. uvarum CBS 7001	wild type	Westerdijk Institute The Netherlands
Weihenstephan 34/70	wild type	Hefebank Weihenstephan, Germany
E1	wild type	Erbslöh Geisenheim GmbH, Germany
S. carlsbergensis CBS 1513	wild type	Westerdijk Institute, The Netherlands
G166-G168	*fot1-fot2::YES1 fot1-fot2::YES3*	Derivatives of E1, this study
Lalvin EC1118 *S. cerevisiae*	wild type	Lallemand Inc., Canada

Table S2. Yeast genomes used during analyses.

Strain	Species	GenBank accession
E1	*Saccharomyces uvarum*	PRJNA970106 (BioProject)
CBS 7001	*S. uvarum*	GCA_019953615.1
FM1318	*S. eubayanus*	GCF_001298625.1
WS 34/70	*S. pastorianus*	GCA_001515485.2
CBS 1483	*S. pastorianus*	GCA_011022315.1
CBS 1513	*S. carlsbergensis*	GCA_001515445.2
CBS 1503	*S. monacensis*	GCA_013183815.1
IFO1802	*S. kudriavzevii*	GCA_000167075.2
CR85	*S. kudriavzevii*	GCA_003327635.1
FM1318	*S. eubayanus*	GCF_001298625.1
UCD646	*S. eubayanus*	GCA_946408725.1
UCD650	*S. eubayanus*	GCA_946405395.1
CEG	*S. cerevisiae × S. kudriavzevii*	JARXNF000000000
EC1118	*S. cerevisiae*	GCA_000218975.1
VIN7	*S. cerevisiae × S. kudriavzevii*	GCA_000326105.1
CLIB 830	*Torulaspora microellipsoides*	LN811465, GCA_900186055.1

Table S3. Primers used in this study.

Number	Name	Sequence*
356	G3-*YES1*	GTGTCGGTATCGCAGAC
604	5′-*FOT1*	TTAGGCCACAGGAGAAG
605	*3′-FOT1*	ATGTCAAAACTTGTCCCTATCG
606	5′-*FOT2*	TCAGGCCACAGGAGAAG
607	3′-*FOT2*	ATGTCAAAGCTCATCCCTATCG
609	G4-*FOT1/2*	CCGTATATGCTCGAGGATC
633	S1-YES	ATATTAGTTATATTTTATAGATCTGCTTGCCATCTT-GCACAATATATCCATTCAAGTCCCTCACCATGGGATTTCCGTCGTCCTGTGATGGCAACTGCGAAGCTTCGTACGCTGCAGGTC
634	S2-YES	AAGGACAGATACCTCAATTTCAAATAGAAACTATAGTTCTT-GCTTAACGATAACAGTC-TATTCCATTTGACTGAAGAAAAAAAAAATTTATTACTGATCTGATATCATCGATGAATTCGAG
748	G3-*YES3*	GTTCCTGCTTGTAATTTGTATG

*sequences underlined correspond to the annealing regions used to amplify the *YES1* or *YES3* markers.
