# Supplementary material for: Mosaic Genome of a British Cider Yeast
Source: Int J Mol Sci. 2023 Jul 7;24(13):11232. doi: 10.3390/ijms241311232 (PMC10342233; doi:10.3390/ijms241311232)
Supplement: Supplementary file 1 [file ijms-24-11232-s001.zip › Supplementary Note Bioinformatics - v2.pdf]

Detailed Methods Bioinformatics

After an initial screening of *S. uvarum* E1 genome content using Nucleotide-Nucleotide BLAST 2.9.0+ [1] against a local database comprising a selection of publicly available yeast genomes, E1 draft genome contigs were first ordered and then re-aligned against a concatenated reference sequence containing the full genome of *S. uvarum* CBS 7001 (GCA_019953615.1) and, additionally individual chromosomes *SeCHRII*, *SeCHRXIV* (both from assembly *S. eubayanus* FM1318, GCF_001298625.1), contig FYBL01000004.1, which contains region C, from *Torulaspora microellipsoides* CLIB 830 (GCA_900186055.1) and chromosomes *SkCHRIX* and *SkCHRXIV* (*S. kudriavzevii* CR85, GCA_003327635.1). Ordering of contigs and subsequent re-alignment were conducted by running MauveContigMover first and then aligning the ordered contigs to the reference using ProgressiveMauve (PM) algorithm from Mauve Multiple Genome Alignment version 2015-02-26 build 10 [2]. The PM alignment was parsed using custom scripts in R version 4.0.3 [3].

Figure 1

This *circos graph* was generated using basic functions from R package *circlize* [4]. Events, such as boundaries of introgression events and non-reciprocal translocations were recovered from the PM alignment and were highlighted on the graph.

Fold read depth was obtained from mapping short reads "back" to the E1 draft assembly and then binning aligned reads in consecutive windows (1 kb) based on a minimum required overlap; we filtered multimapping reads.

Read Mapping was performed by bbmap.sh from the BBTools software suite, version 38.84 [5]. BBmap.sh was executed in global alignment mode (local=f), using the very slow macro (vslow) with a small kmer length (k=9), but with a high minimum alignment score (minid=96), ambiguous reads were mapped to the best matching site (ambiguous=best) and reads were mapped only if both mates mapped properly (killbadpairs=t). Read binning was performed using bedtools version 2.29.0 [6]. Minimum required overlap of a read with a window was 0.67 x read length (-f 0.67). Additional processing of intermediary files was performed with samtools 1.12 [7]. Fold read depth was calculated by dividing the number of reads assigned to any window by the global median number of reads per window.

The *pairwise similarity track* was calculated from the aligned E1 draft assembly (PM alignment) for consecutive windows (1 kb) using function dist.dna() from R package *ape* [8] with arguments model = "TN93" (Tamura *et* Nei 1993 two parameter model; we did not apply γ correction) and pairwise.deletion = TRUE (filters sites containing alignment gaps). The procedure was implemented via custom R scripts. To avoid inflation of sequence divergence at, for example, difficult-to-align sites or near contig ends, we filtered windows with < 900 aligned DNA bases.

The Locally Collinear Blocks (LCBs) track shows all LCBs on a color gradient as they were established on the reference sequences during PM alignment. The alignment produced 21 LCBs. The color gradient is more or less smooth on the E1 draft genome due to the ordering

of E1 contigs prior to PM alignment (We did not intent for individual LCBs to be distinguished by looking at the figure). However, notice breaks in LCB color sequence on the E1 draft genome side (left-hand side) at non-reciprocal translocations (highlighted by increased color saturation). Additionally, segments corresponding to introgression events are highlighted on the LCB track and, on the E1 draft genome (left-hand side of the circos), were excavated for improved visibility.

Figure 2

Figure 2 was created by parsing the ProgressiveMauve output in a similar fashion as described for Figure 1. Contigs were assigned to a reference chromosome according to the number of aligned bases by applying a simple majority rule (i.e. > 50 % of contig length). The thus classified contigs were then plotted in sequence for each chromosome producing bars with vertical lines showing contig boundaries. Introgression events were represented at their respective locations. Non-reciprocal translocations were not shown to avoid cluttering of lines. For the latter reference the circos graph (Figure 1) instead. Figure 2 was generated using custom scripts and base graphics from R.

Figure 3

These violin plots simply summarize the data from the pairwise similarity track of Figure 1. Violin plots were generated using ggplot2 [9].

Figure 4

To further investigate the origin of the introgressed *S. eubayanus* DNA, we aligned the E1 102 kb *SeCHRXIV* segment and flanking regions (just below 10 kb on either side) to several lager yeast genomes and *S. uvarum* CBS 7001 (*cf.* tables 2 and 3 and table S2 for strain IDs) using the Progressive Mauve algorithm from the Mauve PlugIn version 1.13 for Geneious Prime 23.0.4 (Biomatters, Inc., Ackland, New Zealand). Pairwise similarity among sequences was calculated as described above (Figure 1).

Panels A and B illustrate ancestry of the 102 kb and flanking regions: When comparing the DNA sequence of the E1 *CHRXIV* 102 kb segment (incl. flanking regions) to *S. carlsbergensis* CBS 1513 (panel A), accumulation of mismatches occurs on the sequence flanks (*S. uvarum* ancestry) but not along the insert (*S. eubayanus* ancestry). Vice versa, when comparing the segment to *S. uvarum* CBS 7001 (panel B), mismatches do not accumulate at the flanks (*S. uvarum* ancestry) but along the insert (*S. eubayanus* ancestry). Thus, the E1 *CHRXIV* segment comprises of *S. uvarum* flanks with a *S. eubayanus* insert. Panel C simply displays sequence divergence between CBS 1513 and the other strains at greater detail and highlights the divergence of isolates UCD646 and UCD650.

Figure 5

Figure 5 is just an illustration of region C extent and insertion in E1 and EC1118. It was created after aligning the E1 contig containing region C and the adjacent *SuCHRX* DNA sequence, and the EC1118 (GCA_000218975.1) contig containing region C to *Torulaspora*

microellipsoides CLIB 830 (GCA_900186055.1). Alignment was performed using the Progressive Mauve algorithm from the Mauve PlugIn version 1.13 for Geneious Prime 23.0.4 (Biomatters, Inc., Ackland, New Zealand). The graph was generated base graphics from R.

Additional References

1. Camacho, C.; Coulouris, G.; Avagyan, V.; Ma, N.; Papadopoulos, J.; Bealer, K.; Madden, T. L., BLAST+: architecture and applications. *BMC Bioinformatics* **2008**, 10:421.
2. Darling, A. E.; Mau, B.; Perna, N. T., progressiveMauve: Multiple Genome Alignment with Gene Gain, Loss and Rearrangement. *PLOS ONE* **2010**, 5(6): e11147. https://doi.org/10.1371/journal.pone.0011147
3. R Core Team, R: A Language and Environment for Statistical Computing. R Foundation for Statistical Computing, Vienna, 2020. https://www.R-project.org/
4. Gu, Z.; Gu, L.; Eils, R.; Schlesner, M.; Brors, B., Circlize implements and enhances circular visualization in R. *Bioinformatics* **2014**, 30(19): 2811-2812.
5. Bushnell, B.; Rood, J.; Singer, E., BBMerge - Accurate paired shotgun read merging via overlap. *PLoS One* **2017,** 12(10), e0185056.
6. Quinlan A. R.; Hall I.M., BEDTools: a flexible suite of utilities for comparing genomic features. *Bioinformatics* 26(6): 841–842, **2010** https://doi.org/10.1093/bioinformatics/btq033Samtools
7. Danecek, P.; Bonfield, J. K.; Liddle, J.; Marshall, J.; Ohan, V.; Pollard, M. O.; Whitwham, A.; Keane, T.; McCarthy, S. A.; Davies, R. M.; Li, H.; Twelve years of SAMtools and BCFtools.
   GigaScience **2021**, 10(2): giab008, https://doi.org/10.1093/gigascience/giab008
8. Paradis, E.; Schliep, K., ape 5.0: an environment for modern phylogenetics and evolutionary analyses in R. *Bioinformatics* **2019**, 35: 526-528.
9. Wickham, H., ggplot2: Elegant Graphics for Data Analysis, Springer-Verlag New York, 2016, https://ggplot2.tidyverse.org
